# Overexpression of cathepsin K in mice decreases collagen deposition and lung resistance in response to bleomycin-induced pulmonary fibrosis

**DOI:** 10.1186/1465-9921-9-54

**Published:** 2008-07-18

**Authors:** Mrigank Srivastava, Kathrin Steinwede, Riku Kiviranta, Jukka Morko, Heinz-Gerd Hoymann, Florian Länger, Frank Buhling, Tobias Welte, Ulrich A Maus

**Affiliations:** 1 Department of Pulmonary Medicine, Laboratory for Experimental Lung Research, Hannover School of Medicine, Hannover, Germany; 2 Department of Medical Biochemistry and Molecular Biology, University of Turku, Turku, Finland; 3 Division of Pre-clinical Airway Research, Fraunhofer Institute of Toxicology and Experimental Medicine, Hannover, Germany; 4 Department of Pathology, Hannover School of Medicine, Hannover, Germany; 5 Institute of Clinical Chemistry and Laboratory Diagnostics, Carl-Thiem-Klinikum, Cottbus, Germany

## Abstract

**Background:**

Lung fibrosis is a devastating pulmonary disorder characterized by alveolar epithelial injury, extracellular matrix deposition and scar tissue formation. Due to its potent collagenolytic activity, cathepsin K, a lysosomal cysteine protease is an interesting target molecule with therapeutic potential to attenuate bleomycin-induced pulmonary fibrosis in mice. We here tested the hypothesis that over-expression of cathepsin K in the lungs of mice is protective in bleomycin-induced pulmonary fibrosis.

**Methods:**

Wild-type and cathepsin K overexpressing (cathepsin K transgenic; cath K tg) mice were challenged intratracheally with bleomycin and sacrificed at 1, 2, 3 and 4 weeks post-treatment followed by determination of lung fibrosis by estimating lung collagen content, lung histopathology, leukocytic infiltrates and lung function. In addition, changes in cathepsin K protein levels in the lung were determined by immunohistochemistry, real time RT-PCR and western blotting.

**Results:**

Cathepsin K protein levels were strongly increased in alveolar macrophages and lung parenchymal tissue of mock-treated cathepsin K transgenic (cath K tg) mice relative to wild-type mice and further increased particularly in cath K tg but also wild-type mice in response to bleomycin. Moreover, cath K tg mice responded with a lower collagen deposition in their lungs, which was accompanied by a significantly lower lung resistance (R_L_) compared to bleomycin-treated wild-type mice. In addition, cath K tg mice responded with a lower degree of lung fibrosis than wild-type mice, a process that was found to be independent of inflammatory leukocyte mobilization in response to bleomycin challenge.

**Conclusion:**

Over-expression of cathepsin K reduced lung collagen deposition and improved lung function parameters in the lungs of transgenic mice, thereby providing at least partial protection against bleomycin-induced lung fibrosis.

## Background

Lung fibrosis is an unrelenting pulmonary disease afflicting more than 5 million patients worldwide [1]. Patients diagnosed with pulmonary fibrosis have an average survival of < 2 yr from time of diagnosis [2,3]. The disease process is characterized by alveolar epithelial cell injury and hyperplasia, inflammatory cell accumulation, fibroblast hyperplasia, formation of honeycomb cysts and exuberant production and deposition of extracellular matrix (ECM) components, particularly collagen and fibronectin along with scar tissue formation [2,4]. Until now, anti-inflammatory agents and immune modulators have proved to be minimally effective in limiting alveolitis and modifying the course of the disease, thus underlining the urgent need for novel therapeutic intervention strategies.

Cathepsin K (Cath K) is a member of the lysosomal cysteine and aspartic proteinase family, and has been shown to possess unique collagenolytic activities [5]. Cathepsin K is expressed in bone osteclasts and chondroclasts, but also in epithelioid cells and multinucleated giant cells in lung granulomatous lesions, whereas it is largely absent in resident alveolar macrophages of the lung under baseline conditions [6]. In addition, cath K was observed in lung fibroblasts and bronchial epithelium in normal adult lung tissue [6,7]. Recent reports from our group showed that mice deficient of cathepsin K responded with a significantly increased extracellular matrix deposition to challenge with bleomycin when compared to wild-type mice with a normal cath K expression, thus for the first time suggesting a protective role of cathepsin K in lung matrix homeostasis under physiological and pathological conditions [8]. Based on these observations, we hypothesized that over-expression of cathepsin K in the lungs of mice would ameliorate lung fibrosis and lung resistance in mice challenged with bleomycin. To test this hypothesis, cathepsin K transgenic mice were challenged with bleomycin and their lung cell- and tissue-specific cathepsin K expression, lung fibrosis, and lung function was compared with that of bleomycin-treated wild-type mice.

## Methods

### Animals

Cathepsin K transgenic (cath K tg) mice on a FVB/N background were generated as described recently [9]. For all the experiments, we used homozygote siblings (cathepsin K transgenic) derived from matings of heterozygous cath K transgenic mice that were identified by genotyping according to established real-time PCR protocols [10], using genomic DNA extracts from tail snips of the respective mice. Corresponding genotyping results were further confirmed by southern blot analysis (data not shown). Experimental animals were 8–12 weeks old and were used in accordance with the guidelines of our Institutional Animal Care and Use Committee. All experiments involving animals were approved by our local government authorities.

### Reagents

Bleomycin was purchased from Medac (Hamburg, Germany). Hydroxyproline was purchased from Sigma (Deisenhofen, Germany). Mononclonal anti-cathepsin K antibody was purchased from Calbiochem (via EMD Biosciences, Darmstadt, Germany). Fetal calf serum (FCS) and RPMI 1640 medium were purchased from PAA laboratories (Pasching, Austria). Roti-Quick kit for total RNA isolation was purchased from Carl Roth (Karlsruhe, Germany). SYBR green 1 kit for real time RT-PCR was purchased from Eurogentec (Seraing, Belgium). Collagenase A was purchased from Roche (Mannheim, Germany). DNase 1 was purchased from Serva (Heidelberg, Germany). Random hexamer primers, MMLV-reverse transcriptase, recombinant RNase inhibitor, dNTPs and DTT were all purchased from Promega (Madison, US).

### Bleomycin application

Intratracheal application of bleomycin in cath K tg and wild-type mice was done essentially as described earlier [8,11]. Briefly, tracheas of anaesthetized mice were surgically exposed and an Abbocath catheter (Abbott, Wiesbaden, Germany) was inserted into the trachea. Subsequently, 0.025 U of bleomycin diluted in sterile saline or saline only was slowly instilled under streomicroscopic control (Leica MS5, Wetzlar, Germany). After instillations, the skin was sutured and mice were allowed to recover with free access to food and water.

### Collagen content measurements

Wild-type mice and cath K tg mice were either instilled saline or 0.025 U of bleomycin and then euthanized at various time points (1, 2, 3, and 4 weeks) post-treatment. Subsequently, lungs were harvested and assayed for their collagen content by hydroxyproline dye binding assays according to published protocols [12], with some modifications. Briefly, for hydroxyproline measurements, lungs were perfused with saline containing 0.1 M heparin until the lung tissue appeared visually free of blood. While avoiding contaminations with conducting airways and lymphatic tissue, lung lobes were excised, cut into small pieces and homogenized in sterile distilled water followed by overnight hydrolysis in 6N hydrochloric acid at 110°C. Next day, hydrolyzed samples were neutralized in 5N NaOH and dried overnight using a speedvac set at 45°C. For quantification of the collagen content, dried samples were suspended in a small volume of sterile distilled water and thoroughly vortexed followed by addition of assay buffer and chloramine T-reagent. After a brief incubation, DMBA reagent was added and samples were further incubated for 20 min at 60°C. Total collagen content was finally estimated by measuring the absorbance at 570 nm against a reference wavelength of 650 nm and co-relating them against a hydroxyproline standard.

### Collection of bronchoalveolar lavage fluid (BALF)

Bronchoalveolar lavage fluid was collected from saline or bleomycin-treated wild-type and cath K tg mice after 1, 2, 3 and 4 weeks post bleomycin instillation according to recently published protocols [10,11,13-15]. Total cell numbers recovered by BAL were counted with viabilities as determined by trypan blue dye exclusion always exceeding >90%. BAL fluid aliquots were cytospun onto glass slides (Shandon, UK) and subjected to Pappenheim staining for the differentiation and quantification of BAL fluid cellular constituents. In separate experiments, to investigate whether cathepsin K protein would also be contained in BAL fluids of mice of the respective treatment groups, BAL supernatants from wild-type mice or cath K tg mice treated with bleomycin for two weeks were collected and concentrated using VIVAspin 6 centrifugal concentrators (Sartorius, Göttingen, Germany), and these samples were used for the determination of extracellular cathepsin K protein content by western blotting and gelatin zymography.

### Isolation of primary type II alveolar epithelial cells

Primary type II alveolar epithelial cells (AEC) were isolated from saline or bleomycin treated wild-type and cath K tg mice after two weeks of bleomycin challenge according to recently described protocols, with some modifications [16,17]. Briefly, mice were euthanized with an overdose of isoflurane and exsanguinated by cutting the inferior vena cava. Lungs were perfused gently with 10 ml of sterile HBSS via the right ventricle until they were visually free of blood. A small incision was made into the exposed trachea and a shortened 21-gauge cannula was inserted and firmly fixed with a suture. Sterile dispase (approx. 1 ml; BD Biosciences) followed by 500 μl of sterile 1% low-melting agarose in PBS^-/- ^(Sigma-Aldrich) was gently instilled into the lungs. After a short incubation, the lungs were removed and placed into a culture tube containing 2 ml of dispase and incubated for 40 min at room temperature. Lungs were then transferred into a culture dish containing DMEM/2,5% HEPES buffer/0.01% DNase (Serva), and the tissue was carefully dissected from the airways and large vessels. The resultant cell suspension was successively filtered, re-suspended in 10 ml of DMEM supplemented with 10% FCS and antibiotics, and incubated with biotinylated rat anti-mouse CD16/32 and rat anti-mouse CD45 mAbs (BD Pharmingen) for 30 min at 37°C. Cells were then washed and incubated with streptavidin-conjugated MagneSphere Paramagnetic Particles (Promega) for 30 min at room temperature with gentle rocking, followed by magnetic removal of contaminating leukocytes. Purities of freshly isolated AEC was always >90%, as assessed by modified Papanicolaou staining specific for type II AEC. Viabilities of isolated AEC were always >95%, as assessed by trypan blue dye exclusion. Primary AEC were lysed either in Guanidine Isothiocyanate (GITC) or protein lysis buffer for subsequent real-time RT-PCR or western blot analysis, respectively.

### Preparation of lung homogenate

Lungs were perfused with HBSS supplemented with heparin. Thereafter, removed lungs were cut into small pieces and homogenized in 3 ml of protease inhibitor cocktail. The resulting suspension was passed through 100 μM cell strainer and centrifuged. The resulting cell pellet was washed with 1 ml of ice-cold PBS and centrifuged. The supernatant was discarded and 200 μl of lysis buffer was added to the pellet and incubated over ice for 30 min with intermittent mixing. After incubation, the lysate was centrifuged again at 13,000 rpm for 30 min and the supernatant containing the protein was stored at -80°C until western blot analysis was performed.

### Total cellular RNA isolation, cDNA synthesis and real-time RT-PCR

Total cellular RNA was isolated from BAL cells, freshly isolated alveolar type-II epithelial cells and lung homogenates of saline or bleomycin treated wild-type and cath K tg mice after two weeks of bleomycin challenge using a commercially available RNA isolation kit (Carl Roth), following the instructions of the manufacturer. RNA quantification and purity was determined on an Agilent Bioanalyzer 2100 (Agilent Biosystems) and only those RNA preparations exceeding absorbance ratios of (A_260/280_) > 1.90 were further processed for reverse transcription and real-time RT-PCR analysis, as described recently [10]. For normalization, β-actin was used as the housekeeping gene and mean fold-changes were calculated using the 2^-ΔΔCTCT ^method [18]. Primer sequences for PCR detection of cathepsin K mRNA levels are forward 5'-gggccaggatgaaagttgta-3', and reverse 5'-cactgctctcttcagggctt-3' [19].

### Western blotting

For cell-specific detection of cathepsin K protein in BAL cells, lung homogenates and type II alveolar epithelial cells from wild-type or cath K tg mice, cell lysates were prepared in 100 μl of ice cold lysis buffer and equal amounts of protein (20 μg each) were loaded onto 12.5% SDS-polyacrylamide gels and electrophoretically transferred to PVDF membranes (Roth, Karlsruhe, Germany). Cath K protein was detected with anti-cathepsin K monoclonal Ab detecting the mature form of cathepsin K (molecular weight, 29 kDa) (Calbiochem, Darmstadt, Germany) using chemiluminescence ECL substrate (Amersham, Buckinghamshire, UK). Stripped blots were re-probed with anti-β-actin monoclonal Ab (clone AC-15, Sigma, Munich, Germany).

### Lung histopathology and degree of lung fibrosis

Wild-type mice and cath K tg mice were either mock-treated with saline or received 0.025 U of bleomycin and were then euthanized at various time points (1, 2, 3 and 4 weeks) post bleomycin treatment. Subsequently, lungs were inflated *in situ *with PBS-buffered formaldehyde solution (4.5%, pH 7.0, Roth, Darmstadt, Germany), and then carefully removed and immersed in PBS-buffered formaldehyde solution for at least 24 h at room temperature. Lung tissue samples were paraffin-embedded, and sections of 5 μm were prepared and stained with hematoxylin/eosin (H/E) and Trichrome staining (Elastica-van-Gieson). The degree of lung fibrosis was determined according to the method described by Ashcroft and colleagues [20], employing a numerical scaling system in lung samples ranging from 0 (normal lung) to 8 (total fibrous obliteration of the field). The mean degree of lung fibrosis according to the definitions by Ashcroft et al. [20] was calculated from individual scores of ~15 microscopic fields analyzed per mouse lung.

### Zymography

BAL fluids were concentrated using a spin concentrator (Vivaspin, Sartorius, Göttingen, Germany). Subsequently, 50 μg of total BAL fluid protein was mixed with sample loading buffer (Roth, Deisenhofen, Germany) and zymography was performed essentially as described previously [21] with some modifications. Briefly, protein samples were loaded on 12.5% SDS-polyacrylamid gel containing 0.1% gelatin. After electrophoresis, gels were washed twice in 2.5% Triton X-100, rinsed four times in distilled water and incubated overnight in assay buffer (0.1 M sodium acetate, pH 5.5, 1 mM EDTA, 2 mM DTT [22]. Subsequently, gels were stained with colloidal Coomassie (Roth) according to the manufacturer's instructions to visualize protease activity.

### Immunohistochemistry

Formalin-fixed lung specimen from sham-treated wild-type mice and cath K tg mice were subjected to immunohistochemical analysis, as described recently in detail [8,23]. Briefly, immunostaining was performed on lung sections (5 μm) using monoclonal antibody directed against mouse cathepsin K (dilution 1:50, Calbiochem, Darmstadt, Germany). Biotin-conjugated anti-mouse IgG (H+L), diluted 1:200 from MOM Kit (Vector Laboratories, CA) was used as the secondary antibody. Immunoreactions were visualized using the avidin biotin complex method applying Vectastain ABC peroxidase or alkaline phosphatase kits (Vector Laboratories). 3,3'-Diaminobenzidine was used as the substrate and sections were counter-stained with hemalaun. The specificity of immunostaining was tested by substitution of primary antibodies with irrelevant antibodies. Slides were examined at a × 40 original magnification using an inverted microscope (Zeiss axiovert 200, Oberkochen, Germany) equipped with a digital imaging unit.

### Lung function measurements

For measurements of lung resistance and compliance, wild-type mice and cath K tg mice pre-treated with bleomycin for two weeks were anaesthetized with 1.5% halothane/30% oxygen by inhalation soon after pre-medication with 77 mg/kg propofol given by intraperitoneal injection. At the time when anesthesia was achieved, mice were carefully intubated oro-tracheally under visual control by trans-illumination of the neck. The technique for invasive but repetitive lung function measurement used in this study has been described recently [24,25]. Briefly, two intubated, spontaneously breathing animals were placed in supine position in temperature-controlled body plethysmographs (type 871, HSE-Harvard Apparatus, March-Hugstetten, Germany). The oro-tracheal tube was directly attached to a pneumotachograph (HSE) connected to a differential pressure transducer to determine tidal flow. Trans-pulmonary pressure (PTP) was measured via a water-filled esophagus catheter coupled to a pressure transducer (P75, HSE). Lung resistance (R_L_) and dynamic lung compliance (C_dyn_) were calculated from PTP and tidal flow and volume signals over an entire breath cycle. After reaching steady state conditions, respiratory parameters were continuously recorded and averaged for 5 min using HEM 3.5 software (Notocord, Croissy, France). After measurements, mice were extubated as soon as they began recovering from anesthesia and placed back into their cages with free access to food and water.

### Statistical analysis

All data are presented as mean ± SD. Statistical analysis for all the experiments was done by Student t-test or ANOVA followed by post hoc Dunnett test, where appropriate to determine significant differences between groups. *P *values of < 0.05 were considered to indicate statistically significant differences.

## Results

### Characterization of cathepsin K overexpression in the lungs of mice

**Figure 1:**
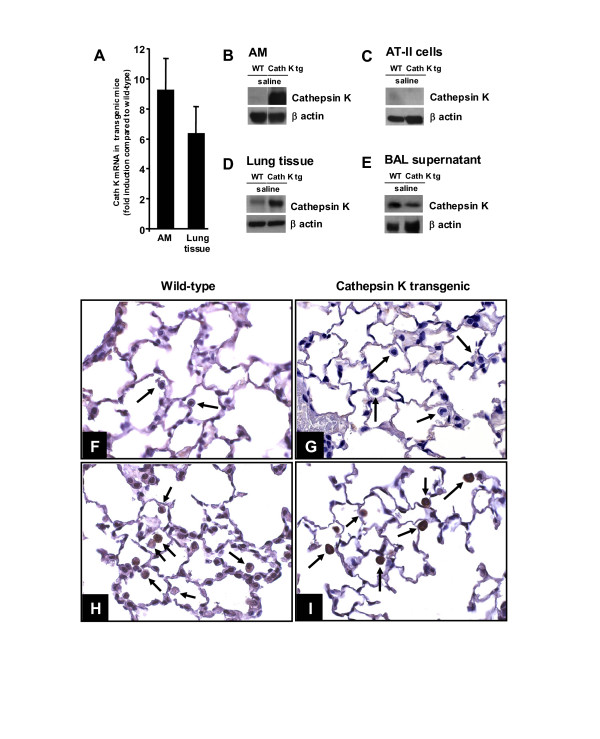
**Characterization of cathepsin K overexpression in the lungs of mice**. Wild-type and cath K tg mice were instilled with saline only (mock-treatment). Twenty-four hours later, cathepsin K mRNA levels were determined in lysates of alveolar macrophages and lung parenchymal tissue of mock-treated cath K tg mice as compared to mock-treated wild-type mice (A). In addition, cathepsin K protein was determined in lysates of alveolar macrophages (B), freshly isolated type II alveolar epithelial cells (C), lung parenchymal tissue (D), and concentrated BAL fluid supernatant (E) of mock-treated cath K transgenic and wild-type mice by western blot analysis. Data represent at least 3–5 independent experiments. (F-I), immunohistochemistry of cath K protein in lung tissue of wild-type mice (F, H) or cath K tg mice (G, I). (F, G) staining with isotype-matched control antibody; (H, I) cathepsin K specific staining. Arrows point to alveolar macrophages *in situ*. Photogmicrographic illustration is representative of at least n = 3 independent analyses. Original magnification, × 40

We initially characterized cathepsin K overexpression in the lungs of transgenic mice compared to wild-type mice. Analysis of cathepsin K mRNA and protein levels in lysates of resident alveolar macrophages and lung homogenates revealed 9.3-fold and 6.4-fold upregulated cathepsin K mRNA levels in alveolar macrophages and lung homogenates, respectively of the transgenic mice as compared to mock-treated wild-type mice (Figure 1A). Also, baseline cathepsin K protein levels were strongly increased in the respective samples collected from cath K overexpressing mice when compared to wild-type mice (Figure 1B, D). In contrast, freshly prepared, primary type II alveolar epithelial cells of both wild-type and cath K tg mice completely lacked cathepsin K protein (Figure 1C). Interestingly, concentrated BAL supernatants of mice of both experimental groups were found to contain cathepsin K protein, suggesting that under baseline conditions, low levels of cathepsin K protein is also released into the bronchoalveolar compartment (Figure 1E). In addition, immunohistochemical analysis of lung sections collected from sham-treated wild-type mice and cath K tg mice revealed low cathepsin K immunoreactivity in alveolar macrophages of wild-type mice and a strongly increased immunoreactivity noted in alveolar macrophages of cathepsin K overexpressing mice (Figure 1F–I).

### Cathepsin K transgenic mice exhibit decreased collagen deposition in their lungs in response to bleomycin compared to wild-type mice

**Figure 2:**
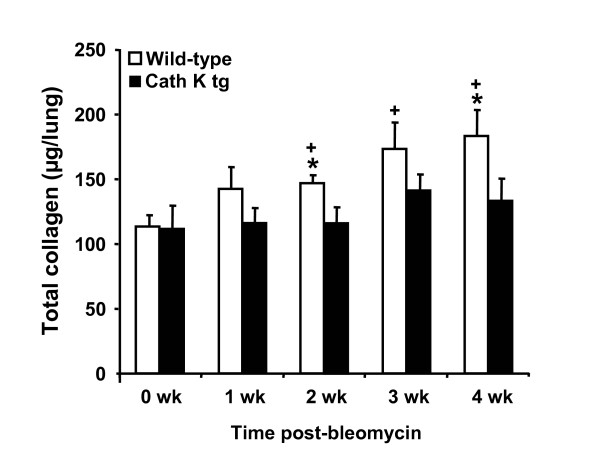
**Cathepsin K transgenic mice exhibit decreased collagen deposition in their lungs in response to bleomycin compared to wild-type mice**. Cathepsin K transgenic mice (black bars) and wild-type mice (white bars) were administered either saline or bleomycin for 1, 2, 3, and 4 weeks. Subsequently, mice were euthanized and lungs were processed for quantitative assessment of collagen content by hydroxyproline assay, as described in Materials and Methods. The data are shown as mean ± SD of at least n = 10 mice per group and time point. * indicates *p *< 0.05 compared to wild-type mice. + indicates significant increase (p < 0.05) compared to mock-treated wild-type mice.

Quantitative measurement of collagen content in the lungs of wild-type mice and cath K tg mice by hydroxyproline assay at various time points post bleomycin challenge showed that transgenic mice exhibiting connatal over-expression of cathepsin K protein in their lungs consistently demonstrated lower total collagen contents in lung parenchymal tissue extracts as compared to wild-type mice with statistically significant differences observed at 2 and 4 weeks post-treatment (Figure 2).

### Effect of bleomycin application on cathepsin K expression in the lungs of mice

**Figure 3:**
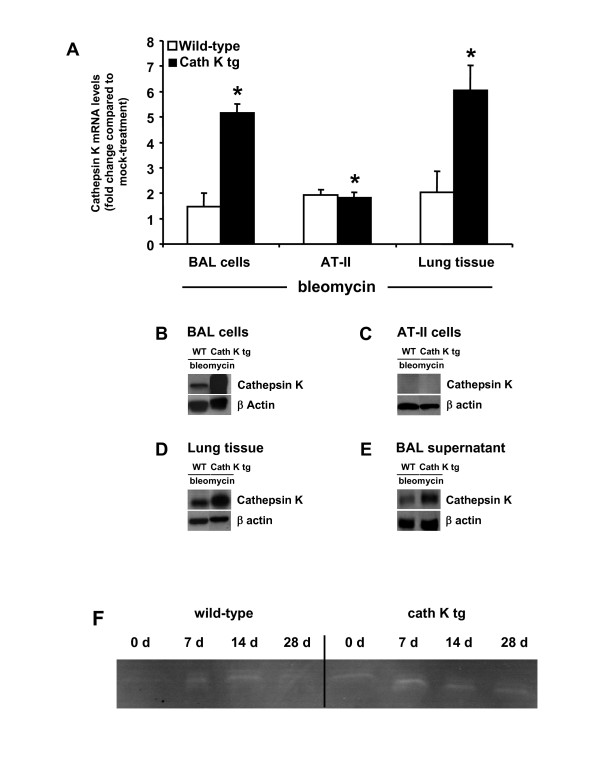
**Effect of bleomycin challenge on cathepsin K expression in the lungs of cath K transgenic and wild-type mice**. Cathepsin K transgenic mice (black bars) and wild-type mice (white bars) were treated with bleomycin for 14 days (A-E). Mice were then euthanized and BAL cells (A, B), freshly isolated type II alveolar epithelial cells (A, C), lung homogenate (A, D), and concentrated BAL fluid supernatant (E) were prepared for analysis of cath K mRNA by real-time RT-PCR (A) and western blotting (B-E). (A), data are given as fold change in cathepsin K mRNA of bleomycin-challenged cath K tg mice compared to wild-type mice. (F) Gelatin zymography employing concentrated BAL fluid protein from mock- or bleomycin-treated wild-type mice and cath K tg mice. Data are shown as mean ± SD of n = 3 determinations. (B-F), data represent at least n = 3–5 independent analyses. * indicates *p *< 0.05 versus bleomycin-treated wild-type mice at day 14 post-treatment.

Our initial results showed that bleomycin-treated cath K tg mice had lower collagen contents in their lungs compared to wild-type mice. Therefore, we questioned whether this effect might be due to further bleomycin-induced upregulation of cathepsin K protein in the transgenic mice as opposed to bleomycin-challenged wild-type mice. Also, an increased bleomycin-triggered inflammatory lung leukocyte recruitment carrying increased cathepsin K protein cargo into the lungs might additionally or alternatively underly the lower collagen contents observed in the transgenic mice. Thus, we determined cathepsin K mRNA and protein levels and proteolytic activity in mice of either treatment group at various time points post-bleomycin. Bleomycin treatment resulted in a significantly increased cath K transcription and protein expression both in alveolar macrophages and lung tissue homogenate of cath K tg mice compared to wild-type mice (Figure 3A,B,D), demonstrating that transgene-dependent increased cath K levels noted under baseline conditions were further upregulated in response to bleomycin challenge. We also observed increased cathepsin K protein contents and proteolytic activities in cell-free BAL fluid concentrates prepared from the lungs of mock-treated and bleomycin-treated cath K tg relative to wild-type mice (Figure 3E, F). On the other hand, no cathepsin K protein was detected in type II epithelial cell lysates of bleomycin-challenged mice of either treatment group at two weeks post-treatment (Figure 3C), confirming and expanding recent reports from our group [8].

**Figure 4:**
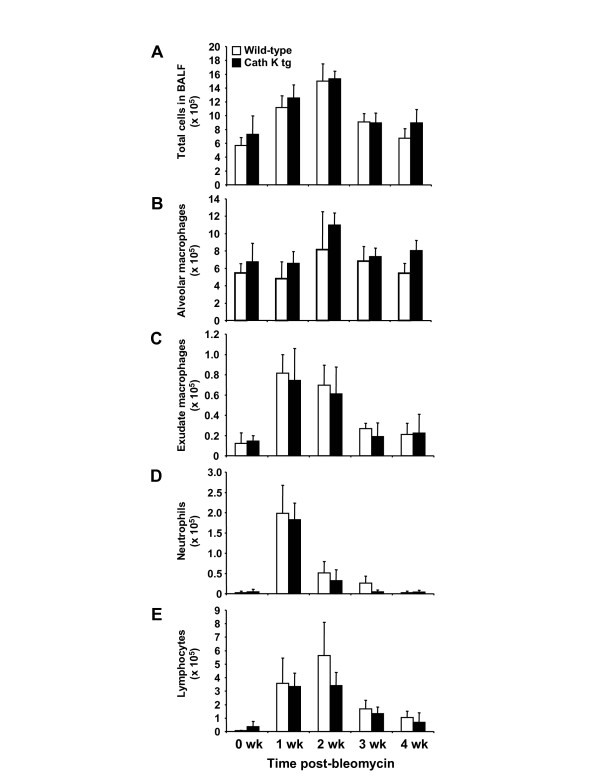
**Effect of bleomycin instillation on leukocyte subset recruitment into the lungs of mice.** Cathepsin K transgenic mice (black bars) and wild-type mice (white bars) were administered either saline or bleomycin for 1, 2, 3, and 4 weeks. Subsequently, mice were euthanized and lungs were lavaged as described in Materials and Methods. Total BAL fluid cell numbers (A), alveolar macrophages (B), exudate macrophages (C), neutrophils (D) and lymphocytes (E) were ascertained from Pappenheim-stained cytospin preparations of BAL fluid. The data are shown as mean ± SD of at least n = 5 mice per group and time point. No statistical significances were noted between wild-type and Cathepsin K transgenic mice.

We next questioned whether differences in inflammatory leukocyte recruitment between cath K tg mice and wild-type mice contributed to differences in lung cathepsin K protein profiles noted in the two experimental groups challenged with bleomycin. However, numbers of alveolar macrophages, exudate macrophages, neutrophils and lymphocytes contained in BAL fluids of mice of either treatment group did not reveal any statistically significant differences between groups, as shown in Figure 4 A-E. These data show that the observed differences in cathepsin K protein profiles were not due to differential inflammatory leukocyte recruitment between groups.

### Bleomycin-induced histopathology in wild-type mice as compared to cathepsin K transgenic mice

**Figure 5:**
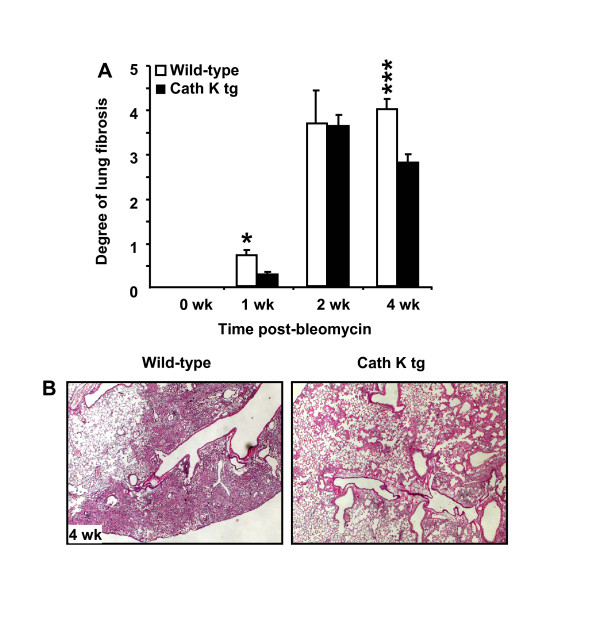
**Degree of lung fibrosis in wild-type mice and cathepsin K transgenic mice**. **(A) **Cathepsin K transgenic mice (black bars) and wild-type mice (white bars) were administered either saline or bleomycin for 1, 2, and 4 weeks. Subsequently, mice were euthanized and lungs were examined histopathologically as described in Materials and Methods and the degree of lung fibrosis per mouse lung was calculated according to the grading system described by Ashcroft et al. [20]. **(B) **Representative photomicrographs of Elastica van Gieson-stained lung tissue sections collected from wild-type mice and cath K tg mice at day 28 post-bleomycin treatment. The data are shown as mean ± SD of at least n = 6 mice per group and time point. * indicates significant increase (*p *< 0.05) compared to cathepsin K tg mice.

Histopathological analysis of lung sections from mock-treated wild-type mice or cathepsin K tg mice displayed a similar lung architecture with no differences noted between groups (data not shown). By one week post-bleomycin treatment, wild-type mice and cathepsin K transgenic mice were found to develop early perivascular and peribronchial lymphohistiocytic infiltrates with significantly increased lung fibrosis developing in the wild-type but not cath K tg mice (Figure 5). At two and four weeks post-treatment, both wild-type mice and cath K tg mice demonstrated epithelial hyperplasia of terminal bronchioles and surrounding alveoli and lymphohistiocytic infiltration of the alveolar septae with fibroblast proliferation. Importantly, the degree of lung fibrosis according to the socring system developed by Ashcroft et al. [20] was significantly higher in wild-type mice relative to cath K tg mice as early as one week post-bleomycin and even more pronounced by four weeks post bleomycin challenge. Figure 5B shows representative trichrome (Elastica-van-Gieson)-stained lung tissue sections from mice of the corresponding treatment groups, thereby illustrating the differences in developing lung fibrosis between groups by four weeks post-bleomycin challenge. These data demonstrate that cathepsin K over-expression attenuates, at least in part, lung histopathological manifestations developing in response to bleomycin challenge (Figure 5).

### Cathepsin K transgenic mice exhibit reduced lung resistance upon bleomycin challenge

**Figure 6:**
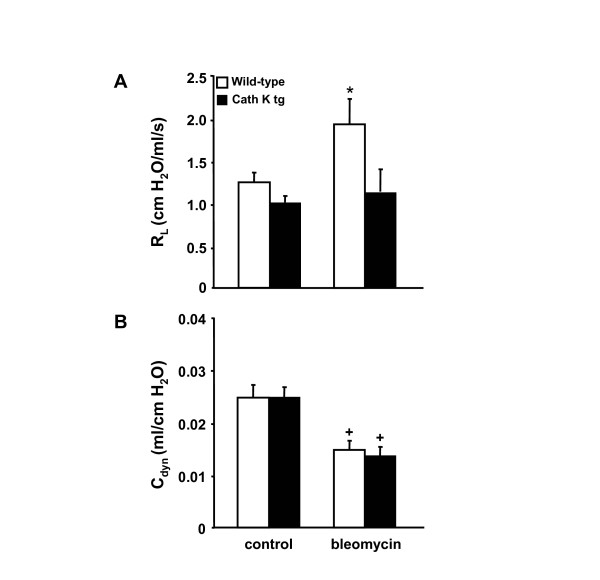
**Cathepsin K transgenic mice exhibit decreased lung resistance upon bleomycin challenge**. Cathepsin K transgenic mice (black bars) and wild-type mice (white bars) were treated with bleomycin for 14 days. Subsequently, mice were anaesthesized and their lung resistance (A) and dynamic compliance (B) were assessed and compared to controls (A, B). The data are shown as mean ± SD of at least n = 10 mice per group and time point. * indicates significant increase (*p *< 0.05) compared to cathepsin K transgenic mice. + indicates significant decrease (*p *< 0.05) compared to the respective mock-treated mice.

Lung resistance (R_L_) and dynamic compliance (C_dyn_) are important end-points in the determination of pulmonary mechanics [26]. Therefore, we determined lung resistance and dynamic compliance in wild-type mice and cathepsin K transgenic mice at 2 weeks post bleomycin application. As shown in Figure 6, cathepsin K transgenic mice had a similar lung resistance compared to wild-type mice under baseline conditions (mock-treatment) (Figure 6A). However, in response to bleomycin challenge, lung resistance significantly increased in the wild-type mice only but not in the cath K transgenic mice, while at the same time, dynamic compliance was significantly reduced in mice of either treatment group challenged with bleomycin (Figure 6B).

## Discussion

In the current study, we show that cathepsin K overexpressing mice were partially protected from bleomycin-induced pulmonary fibrosis compared to wild-type mice exhibiting a normal cathepsin K expression in their lungs, as assessed by partially reduced lung collagen contents and lack of increasing lung resistance in response to bleomycin challenge. As such, the currently presented data add to our recent report on the role of cathepsin K in contributing to collagen turnover in the lungs of mice.

Pulmonary fibrosis is a major clinical problem characterized by accumulation of extracellular matrix (ECM) proteins, including collagen, fibronectin, proteoglycans, and elastin, leading to abnormal lung architecture and severe impairment of lung function [27]. There is currently no therapy for pulmonary fibrosis or other fibrosing diseases, leading to unacceptably high morbidity and mortality rates among affected individuals [27,28]. Matrix metalloproteases (MMPs) have long been known to exert a myriad of regulatory actions critical in tissue repair and remodelling, including epithelial cell migration, proliferation, differentiation, and apoptosis, as well as release of latent or bound growth factors from the ECM [29]. However, over the past few years, lysosomal proteases such as the cysteine protease cathepsin K have been linked with matrix turnover and thus gained increasing attention [30,31]. Cathepsin K is the most potent mammalian elastase yet described, as it possesses a unique collagenolytic activity among other proteases [5,32]. Recent reports from our group showed that cathepsin K plays a pivotal role during bleomycin-induced lung fibrosis [8]. More specifically, we found that cathepsin K knockout mice (CTSK^-/-^) deposited significantly more extracellular matrix upon bleomycin challenge than control mice, and primary lung fibroblasts derived from these animals showed a decreased collagenolytic activity, thus supporting a role for cathepsin K in lung collagen degradation. In the present study, we found that global overexpression of cathepsin K effected a partially increased extracellular matrix turnover in the lungs of mice challenged with bleomycin. At the same time, an increased expression of cathepsin K particularly in alveolar macrophages and lung tissue homogenate but not in type II alveolar epithelial cells both under baseline conditions and in response to bleomycin was noted, suggesting that alveolar macrophages but not type II alveolar epithelial cells contributed, at least in part, to the increased collagenolytic activities in the transgenic as opposed to wild-type mice challenged with bleomycin. In the current study, cathepsin K was not detected in freshly isolated type II alveolar epithelial cells, as opposed to bronchial epithelial cells previously reported to express cathepsin K [7]. Thus, as opposed to human or murine lung fibroblasts [8] or human bronchial epithelial cells [7], type II epithelial cells do not appear to contribute to cathepsin K-dependent collagenolytic activities in bleomycin-induced lung fibrosis.

Since in the current study, numbers of BAL fluid cell differentials were similar in the transgenic mice compared to wild-type mice upon bleomycin treatment, the data suggest that primarily the transgene-induced increased cathepsin K translation in alveolar macrophages rather than differential leukocyte mobilization towards the lungs of mice *per se *was responsible for the reduced collagen deposition in the transgenic as compared to wild-type mice challenged with bleomycin.

Lung resistance (R_L_) significantly increased in the wild-type mice but not in the cath K overexpressing mice in response to bleomycin challenge, whereas the classical parameter to determine changes in lung elasticity due to fibrosis, i.e. lung compliance [33], was not different between groups. In the present study, lung resistance (R_L_) was determined from the ratio of transpulmonary pressure (PTP) to tidal flow over an entire breath cycle [25,26]. Of note, the parameter lung resistance includes airway resistance but also lung tissue resistance. In normal rodents, lung resistance nearly equals airway resistance [33]. In restrictive airway diseases such as pulmonary fibrosis, lung tissue resistance may become more important and significantly contributes to R_L _and thus may have contributed to the effects observed in the current study. In addition, obstruction of peripheral airways also contributes to an increase in R_L_. Fibrosis is also thought to cause airway obstruction mainly in peripheral airways, together with the changes in structural rigidity of lung parenchymal tissue due to lung interstitial inflammation and fibrosis resulting in decreased elastic recoil [24,25]. As such, the currently presented data suggest that cathepsin K overexpression in mice may at least partially prevent changes in lung function evoked by bleomycin treatment.

One unresolved question of the current study is the identification of the mechanism by which bleomycin challenge further increased cathepsin K mRNA and protein levels both in wild-type mice and in transgenic mice. A more precise understanding of the factors governing cathepsin K regulation *in vivo *would be particularly important in view of future strategies to regulate its activity in fibrosing human lungs. Current experiments are being performed to address this unresolved question.

## Conclusion

Together, this study shows that overexpression of cathepsin K does not adversely affect normal lung architecture in mice but at least partially protects against peak lung fibrotic responses and lung resistance upon bleomycin challenge. These data suggest a protective role of increased cathepsin K levels to antagonize excessive ECM deposition in diseased lungs.

## Competing interests

The authors declare that they have no competing interests.

## Authors' contributions

MS and KS carried out the experimental studies and drafted the manuscript. RK and JM provided the transgenic mice and helped with genotyping. HGH carried out the lung function measurements. FL performed the histopathological analysis of fibrotic lungs. FB gave inputs for carrying out the study. TW and UAM designed the experimental setup, supervised the experimental work and participated in the manuscript preparation and provided important intellectual content. All authors read and approved the final manuscript.

## References

[B1] Verma S, Slutsky AS (2007). Idiopathic pulmonary fibrosis--new insights. N Engl J Med.

[B2] King TE, Schwarz MI, Brown K, Tooze JA, Colby TV, Waldron JA, Flint A, Thurlbeck W, Cherniack RM (2001). Idiopathic pulmonary fibrosis: relationship between histopathologic features and mortality. Am J Respir Crit Care Med.

[B3] Coultas DB, Zumwalt RE, Black WC, Sobonya RE (1994). The epidemiology of interstitial lung diseases. Am J Respir Crit Care Med.

[B4] Thannickal VJ, Toews GB, White ES, Lynch JP, Martinez FJ (2004). Mechanisms of pulmonary fibrosis. Annu Rev Med.

[B5] Garnero P, Borel O, Byrjalsen I, Ferreras M, Drake FH, McQueney MS, Foged NT, Delmas PD, Delaisse JM (1998). The collagenolytic activity of cathepsin K is unique among mammalian proteinases. J Biol Chem.

[B6] Buhling F, Reisenauer A, Gerber A, Kruger S, Weber E, Bromme D, Roessner A, Ansorge S, Welte T, Rocken C (2001). Cathepsin K--a marker of macrophage differentiation?. J Pathol.

[B7] Buhling F, Waldburg N, Gerber A, Hackel C, Kruger S, Reinhold D, Bromme D, Weber E, Ansorge S, Welte T (2000). Cathepsin K expression in human lung. Adv Exp Med Biol.

[B8] Buhling F, Rocken C, Brasch F, Hartig R, Yasuda Y, Saftig P, Bromme D, Welte T (2004). Pivotal role of cathepsin K in lung fibrosis. Am J Pathol.

[B9] Kiviranta R, Morko J, Uusitalo H, Aro HT, Vuorio E, Rantakokko J (2001). Accelerated turnover of metaphyseal trabecular bone in mice overexpressing cathepsin K. J Bone Miner Res.

[B10] Srivastava M, Jung S, Wilhelm J, Fink L, Buhling F, Welte T, Bohle RM, Seeger W, Lohmeyer J, Maus UA (2005). The inflammatory versus constitutive trafficking of mononuclear phagocytes into the alveolar space of mice is associated with drastic changes in their gene expression profiles. J Immunol.

[B11] Maus U, Huwe J, Maus R, Seeger W, Lohmeyer J (2001). Alveolar JE/MCP-1 and endotoxin synergize to provoke lung cytokine upregulation, sequential neutrophil and monocyte influx, and vascular leakage in mice. Am J Respir Crit Care Med.

[B12] Woessner JF (1961). The determination of hydroxyproline in tissue and protein samples containing small proportions of this imino acid. Arch Biochem Biophys.

[B13] Maus UA, Backi M, Winter C, Srivastava M, Schwarz MK, Ruckle T, Paton JC, Briles D, Mack M, Welte T, Maus R, Bohle RM, Seeger W, Rommel C, Hirsch E, Lohmeyer J, Preissner KT (2007). Importance of phosphoinositide 3-kinase gamma in the host defense against pneumococcal infection. Am J Respir Crit Care Med.

[B14] Srivastava M, Meinders A, Steinwede K, Maus R, Lucke N, Buhling F, Ehlers S, Welte T, Maus UA (2007). Mediator responses of alveolar macrophages and kinetics of mononuclear phagocyte subset recruitment during acute primary and secondary mycobacterial infections in the lungs of mice. Cell Microbiol.

[B15] Winter C, Taut K, Srivastava M, Langer F, Mack M, Briles DE, Paton JC, Maus R, Welte T, Gunn MD, Maus UA (2007). Lung-specific overexpression of CC chemokine ligand (CCL) 2 enhances the host defense to *Streptococcus pneumoniae* infection in mice: role of the CCL2-CCR2 axis. J Immunol.

[B16] Corti M, Brody AR, Harrison JH (1996). Isolation and primary culture of murine alveolar type II cells. Am J Respir Cell Mol Biol.

[B17] Rosseau S, Selhorst J, Wiechmann K, Leissner K, Maus U, Mayer K, Grimminger F, Seeger W, Lohmeyer J (2000). Monocyte migration through the alveolar epithelial barrier: adhesion molecule mechanisms and impact of chemokines. J Immunol.

[B18] Livak KJ, Schmittgen TD (2001). Analysis of relative gene expression data using real-time quantitative PCR and the 2(-Delta Delta C(T)) Method. Methods.

[B19] Schurigt U, Stopfel N, Huckel M, Pfirschke C, Wiederanders B, Brauer R (2005). Local expression of matrix metalloproteinases, cathepsins, and their inhibitors during the development of murine antigen-induced arthritis. Arthritis Res Ther.

[B20] Ashcroft T, Simpson JM, Timbrell V (1988). Simple method of estimating severity of pulmonary fibrosis on a numerical scale. J Clin Pathol.

[B21] Dreier R, Wallace S, Fuchs S, Bruckner P, Grassel S (2001). Paracrine interactions of chondrocytes and macrophages in cartilage degradation: articular chondrocytes provide factors that activate macrophage-derived pro-gelatinase B (pro-MMP-9). J Cell Sci.

[B22] Platt MO, Ankeny RF, Jo H (2006). Laminar shear stress inhibits cathepsin L activity in endothelial cells. Arterioscler Thromb Vasc Biol.

[B23] von Wulffen W, Steinmueller M, Herold S, Marsh LM, Bulau P, Seeger W, Welte T, Lohmeyer J, Maus UA (2007). Lung dendritic cells elicited by Fms-like tyrosine 3-kinase ligand amplify the lung inflammatory response to lipopolysaccharide. Am J Respir Crit Care Med.

[B24] Glaab T, Mitzner W, Braun A, Ernst H, Korolewitz R, Hohlfeld JM, Krug N, Hoymann HG (2004). Repetitive measurements of pulmonary mechanics to inhaled cholinergic challenge in spontaneously breathing mice. J Appl Physiol.

[B25] Hoymann HG (2007). Invasive and noninvasive lung function measurements in rodents. J Pharmacol Toxicol Methods.

[B26] Irvin CG, Bates JH (2003). Measuring the lung function in the mouse: the challenge of size. Respir Res.

[B27] Perez A, Rogers RM, Dauber JH (2003). The prognosis of idiopathic pulmonary fibrosis. Am J Respir Cell Mol Biol.

[B28] Walter N, Collard HR, King TE (2006). Current perspectives on the treatment of idiopathic pulmonary fibrosis. Proc Am Thorac Soc.

[B29] Sternlicht MD, Werb Z (2001). How matrix metalloproteinases regulate cell behavior. Annu Rev Cell Dev Biol.

[B30] Everts V, van der Zee E, Creemers L, Beertsen W (1996). Phagocytosis and intracellular digestion of collagen, its role in turnover and remodelling. Histochem J.

[B31] Bienkowski RS, Gotkin MG (1995). Control of collagen deposition in mammalian lung. Proc Soc Exp Biol Med.

[B32] Chapman HA, Riese RJ, Shi GP (1997). Emerging roles for cysteine proteases in human biology. Annu Rev Physiol.

[B33] Mauderly JL (1995). Assessment of pulmonary function and the effects of inhaled toxicants. McClellan, R O and Henderson, R F (Eds) Concepts in inhalation toxicology Taylor & Francis, Washington DC, London.

